# Towards a Thriving Workplace: A Feasibility Study on Mindfulness and the Mediterranean Lifestyle for Corporate Wellness

**DOI:** 10.3390/healthcare13010009

**Published:** 2024-12-24

**Authors:** Efstratios Christodoulou, Eleni Poutli, Demetriana Andreou, Sotiria Laoutari, Fani Athanasiou, Yiannis Kourkoutas, Antonios E. Koutelidakis

**Affiliations:** 1 Laboratory of Nutritional and Public Health, Department of Food Science and Nutrition, University of the Aegean, 81400 Myrina, Greece; fnsd22002@aegean.gr (E.C.);; 2 Laboratory of Applied Microbiology & Biotechnology, Department of Molecular Biology & Genetics, Democritus University of Thrace, 68100 Alexandroupolis, Greece

**Keywords:** mindfulness, mediterranean lifestyle, corporate wellness, psychological resilience, work ability, job satisfaction, workplace well-being, employee productivity

## Abstract

**Background/Objectives:** This study explores the potential of integrating mindfulness and the Mediterranean lifestyle into corporate wellness programs to enhance workplace well-being. **Methods:** A survey of 485 employees from Greece and Cyprus examined how mindfulness, resilience, adherence to the Mediterranean lifestyle, and work ability are connected. **Results:** Pearson correlation analysis showed statistically significant positive relationships between mindfulness, resilience, and work ability (*p* < 0.001 and *p* < 0.001, respectively). Mindfulness was associated with higher job satisfaction (*p* < 0.001) and was a significant predictor of good (OR = 1.112, 95% CI: 1.043–1.186, *p* = 0.001) and excellent (OR = 1.163, 95% CI: 1.087–1.245, *p* < 0.001) work ability, while adherence to the Mediterranean lifestyle had a lower yet significant positive correlation with resilience (*p* < 0.01) and work ability (*p* < 0.05). Differences in wellness across job sectors were observed: employees in health and retail sectors had lower resilience, work ability, and mindfulness compared to those in manufacturing, technology, education, and services. Notably, 78% of participants expressed interest in future wellness programs, favoring a combination of online and onsite formats. **Conclusions:** These findings suggest that mindfulness and the Mediterranean lifestyle can enhance employee well-being and productivity, but sector-specific strategies may be necessary to address unique challenges. Practical applications include tailoring interventions to meet the needs of employees in sectors with lower wellness scores. Future research should investigate the long-term benefits of such programs across diverse industries and employee groups.

## 1. Introduction

The modern workplace is increasingly recognized as a source of stress, with employees facing high demands, extended working hours, and significant pressure to deliver exceptional performance. These factors have contributed to a rise in stress-related disorders, burnout, and poor overall well-being, posing substantial challenges for both individuals and organizations. In response, there has been a growing interest in workplace interventions aimed at promoting employee wellness and productivity. Among the most promising approaches are mindfulness and lifestyle interventions, particularly those rooted in the Mediterranean lifestyle, both of which have demonstrated the potential to improve mental and physical health outcomes in various contexts [1,2].

Mindfulness, defined as the practice of paying focused attention to the present moment without judgment, has been widely studied in clinical and organizational settings. Research has shown that mindfulness can help reduce stress, enhance emotional regulation, and improve attention and cognitive function, leading to better work performance and overall well-being [3,4]. Mindfulness interventions have been shown to improve mental health by decreasing symptoms of anxiety, depression, and burnout, making them a promising tool for addressing the challenges faced in today’s fast-paced, high-pressure workplaces [5,6]. However, despite the growing body of evidence supporting its benefits, the scalability and effectiveness of mindfulness interventions within corporate environments remain subjects of ongoing debate [7].

The Mediterranean lifestyle, the traditional lifestyle of people in the Mediterranean region, is a holistic approach to well-being, characterized by adherence to the Mediterranean diet, regular physical activity, strong social connections, quality sleep, sustainability, and a close relationship with nature. The Mediterranean diet itself is centered around nutrient-dense, whole foods, including abundant fruits, vegetables, whole grains, legumes, and healthy fats like olive oil, with moderate consumption of fish, poultry, and dairy, and minimal intake of red meat and processed foods. This diet, celebrated for its nutritional balance, has been extensively linked to reduced risks of chronic diseases, improved cognitive function, and enhanced emotional well-being [8,9]. Beyond diet, the Mediterranean lifestyle emphasizes physical activity, whether through intentional exercise or daily movement, and a strong sense of community, which fosters resilience against stress and promotes mental health through social support. High sleep quality, sustainable practices, and proximity to nature are also integral aspects, contributing to a balanced, fulfilling life that supports both individual and environmental health [10].

The holistic nature of the Mediterranean lifestyle, emphasizing community and physical health, may complement the mindfulness focus on mental clarity and stress reduction, offering a synergistic effect that enhances workplace productivity and well-being. By combining these two approaches, organizations may address both the physical and mental dimensions of employee wellness, creating a more comprehensive framework for intervention. Despite these benefits, integrating the Mediterranean lifestyle into corporate wellness programs has been less explored, and its potential effects on employee wellness and productivity in such contexts remain uncertain.

Although research has examined mindfulness and the Mediterranean lifestyle separately, few studies have explored the association of mindfulness and the Mediterranean lifestyle with corporate wellness. The majority of research has focused on isolated interventions, such as mindfulness-based stress reduction (MBSR) or dietary changes, with limited investigation into their combined impact on the workplace [11,12]. Additionally, while existing studies have reported the positive effects of both mindfulness and the Mediterranean lifestyle on individual health outcomes, there is a notable gap in understanding how these interventions might be integrated into corporate settings in a manner that is practical, scalable, and sustainable [13].

This study aims to bridge this gap by exploring the association between mindfulness, the Mediterranean lifestyle, and factors known to influence wellness and productivity within a corporate framework, including psychological resilience, sleep quality, job satisfaction, and work ability [14,15,16,17]. The purpose of this work is twofold: first, to assess the potential benefits of such an integrated approach on employee well-being and productivity, and second, to gather critical knowledge necessary to design a randomized controlled trial (RCT) to rigorously measure the effects of mindfulness and Mediterranean lifestyle interventions on corporate wellness and productivity outcomes. By conducting a feasibility study, we aim to identify the logistical, organizational, and practical challenges involved in implementing these interventions in the workplace and to establish a framework for future, more definitive trials.

Our research hypothesizes that the combined effects of mindfulness and the Mediterranean lifestyle will lead to improved mental and physical health, heightened job satisfaction, and enhanced work ability. We propose that mindfulness, which promotes mental clarity and stress reduction, and the Mediterranean lifestyle, which emphasizes physical health, social connection, and sustainable practices, will have a synergistic effect, providing greater benefits than either intervention alone. Specifically, we aim to investigate how these interventions—both individually and together—affect psychological resilience, sleep quality, job satisfaction, and work ability. Additionally, we will explore their potential integration into workplace wellness programs to maximize employee well-being and productivity. Given that both mindfulness and the Mediterranean lifestyle independently contribute to health outcomes, their combined application may offer a more effective approach to promoting corporate wellness. By providing foundational knowledge for a future randomized controlled trial (RCT), this study aims to offer valuable insights into the development of more effective workplace wellness programs that enhance both individual health and organizational productivity.

## 2. Materials and Methods

### 2.1. Design and Procedure

This study employed a cross-sectional research design using an online survey available in both Greek and English, distributed via the Sogolytics platform [18]. The cross-sectional design was chosen for its practicality in capturing data at a single point in time, which is particularly useful for assessing relationships among variables such as mindfulness, resilience, and adherence to the Mediterranean lifestyle within a specific workforce population. However, a limitation of this design is its inability to infer causality, as it does not capture temporal changes or longitudinal dynamics in the relationships studied. The first three survey questions functioned as inclusion and exclusion criteria, ensuring participants acknowledged the study’s terms, were employed, and between 18 and 65 years of age. The survey was sent through direct messaging across various social media platforms, accompanied by follow-up reminders to maximize participant engagement and provide assistance with any questions or concerns regarding the study. While online surveys are an effective tool for reaching a broad audience, they may introduce selection bias, as individuals with greater internet access or familiarity with technology are more likely to participate. To mitigate this, we distributed the survey across diverse platforms, including those targeted at less tech-savvy populations, and provided straightforward instructions to encourage wide participation [19,20,21].

Prior to data collection, the study’s objectives and hypotheses were defined, creating a structured framework for analyzing and interpreting the findings. The sample, while primarily drawn from Greece and Cyprus, included participants from various professional backgrounds, reflecting some diversity. Nonetheless, it is acknowledged that this sample may not be fully representative of broader populations outside these regions. The cultural and occupational contexts of Greece and Cyprus may limit the generalizability of findings to other countries. To address this limitation, future studies should aim to include more geographically and culturally diverse populations. After applying inclusion and exclusion criteria, a final sample of 485 adult participants was selected for analysis.

### 2.2. Scales

#### 2.2.1. Mindful Attention Awareness Scale (MAAS-15)

The Mindful Attention Awareness Scale (MAAS-15) is a widely used self-report instrument for assessing mindfulness, developed by Kirk Warren Brown and Richard M. Ryan. This 15-item scale focuses on an individual’s awareness and attention in daily life [22]. Unlike scales that emphasize meditation or specific mindfulness exercises, the MAAS-15 measures an individual’s general attention capacity across different activities, making it adaptable to various contexts and populations. With high internal consistency, test-retest reliability, and strong construct validity, the MAAS-15 is recognized as a reliable measure for assessing mindfulness [23]. Additionally, the Greek version of the MAAS-15 has been validated, making it appropriate for diverse cultural backgrounds [24]. In this study, the MAAS-15 demonstrated an internal consistency index (Cronbach’s α) of 0.887, indicating strong reliability.

#### 2.2.2. Mediterranean Lifestyle Index (MEDIFE)

The Mediterranean Lifestyle Index (MEDLIFE) was employed to assess adherence to the Mediterranean lifestyle in this study. This validated tool evaluates not only the intake of specific foods but also traditional Mediterranean lifestyle habits, such as light wine consumption, minimal snacking, regular short naps, adequate nighttime sleep, and social interactions. The index includes 28 items grouped into three categories: food intake frequencies (15 items), Mediterranean dietary habits (7 items), and physical activity, rest, and social habits (6 items). Each item is scored as 0 or 1, yielding a total score ranging from 0 (lowest adherence) to 28 (highest adherence) [25]. In this study, MEDLIFE demonstrated a Cronbach’s alpha of 0.618, indicating acceptable reliability for exploratory research purposes.

#### 2.2.3. Connor–Davidson Resilience Scale—10 Items (CD-RISC-10)

The 10-item Connor–Davidson Resilience Scale (CD-RISC-10) is a widely recognized measure of resilience, capturing an individual’s ability to cope with stress and adversity. It assesses core resilience characteristics such as adaptability, perseverance, and self-efficacy. Developed to measure resilience across different populations, the CD-RISC-10 has demonstrated high reliability and validity in numerous studies, making it suitable for assessing resilience in both clinical and non-clinical settings [26]. Its application spans various fields, including mental health, organizational behavior, and educational research, due to its robust psychometric properties [27]. This scale was included in our study to measure psychological resilience, an essential factor in workplace wellness and productivity. Cronbach’s a = 0.857.

#### 2.2.4. Single Item Sleep Quality Scale (SQS)

The Single Item Sleep Quality Scale (SQS) provides a quick and practical assessment of sleep quality by asking respondents to rate their sleep quality over the past seven days on a scale from 0 to 10. The simplicity of the SQS allows for efficient data collection without compromising sensitivity, as the visual analog format enables respondents to self-assess their sleep quality effectively [28]. The SQS has been validated as a reliable tool for evaluating sleep quality in healthy adult populations and has shown good correlations with more comprehensive multi-item sleep scales, making it a practical choice for large-scale surveys where brevity is essential [29].

#### 2.2.5. Single Item Job Satisfaction Scale

Job satisfaction was measured using a single-item scale in which participants rated their satisfaction with their current job on a four-point Likert scale (satisfied, fairly satisfied, fairly dissatisfied, dissatisfied). Scores were reverse-coded to ensure higher scores reflected greater satisfaction. Adapted from the Brief Job Stress Questionnaire, this item has been widely used in workplace studies and has demonstrated acceptable test-retest reliability, with a correlation coefficient of r = 0.468 over a one-year period [30]. Single-item measures of job satisfaction have been validated against multi-item scales and are generally considered reliable for capturing overall job satisfaction, particularly in settings where survey length must be minimized [31].

#### 2.2.6. Work Ability Index (WAI)

The Work Ability Index (WAI) is a comprehensive tool developed to assess an individual’s capacity to work based on their health status, job demands, and mental resources. Initially developed for occupational health research in Finland, the WAI has been widely adopted internationally and adapted to various work environments and populations [32]. It consists of multiple dimensions, including current work ability, work ability in relation to physical and mental job demands, and personal resources for managing job requirements. The WAI’s robust psychometric properties, including reliability and validity across diverse job types and demographic groups, make it a versatile and valuable measure of work ability in relation to overall health and well-being [33].

### 2.3. Demographics, Anthropometrics, and Lifestyle

To minimize survey dropout rates [34], demographic and anthropometric questions were placed at the end of the survey. Demographic data included education, employment status, marital status, and gender. Participants were asked to report their height and weight for BMI calculation, a method that, despite being self-reported, remains a valid means of estimating BMI across diverse populations [35]. BMI categories (underweight, normal weight, overweight, and obesity) were based on established guidelines [36]. Participants’ lifestyles were assessed through yes-or-no questions regarding proximity to nature, and sustainable nutrition practices such as avoiding food waste and choosing local and seasonal products. Sustainability and connection to nature are recognized as core components of the Mediterranean lifestyle [37]. Questions assessing experience and knowledge in areas such as nutrition, meal preparation, mindfulness, exercise, and sustainable eating were presented on a 0–10 scale. These questions served a dual purpose: first, to identify participants’ lifestyle habits, and second, to explore potential correlations between these habits and outcomes related to physical health, mental well-being, and work ability.

## 3. Results

### 3.1. Demographics

**Figure 1:**
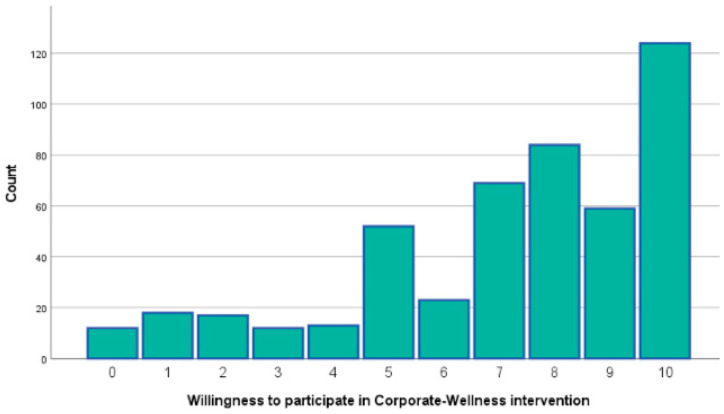
Willingness to participate.

**Figure 2:**
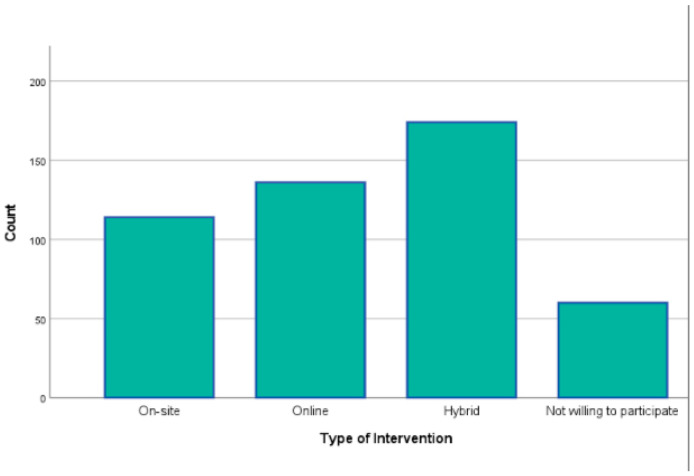
Type of intervention preference.

**Figure 3:**
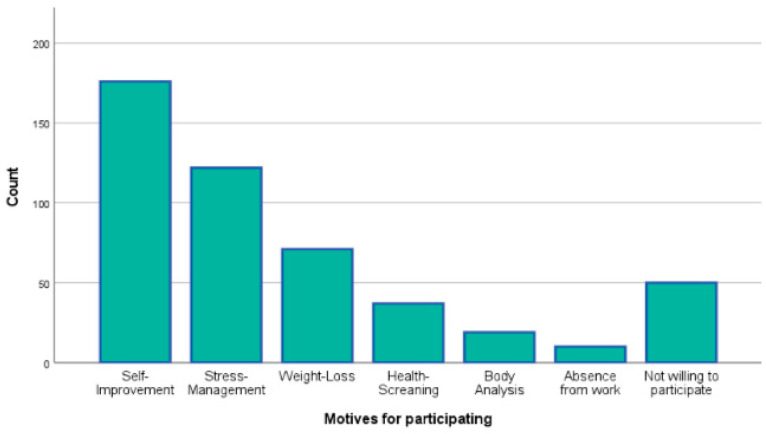
Motives for participating.

The survey initially included 588 participants, with the majority hailing from Greece and Cyprus (95%). A total of 85 individuals who did not meet the inclusion criteria and 18 whose responses were incomplete (fewer than 50% of the total questions answered) or sporadic (survey completed in less than three minutes) were excluded. The final sample comprised 485 workforce individuals aged 18 to 65 years, evenly distributed across four age groups: 18–29 years (27%), 30–39 years (26.8%), 40–49 years (23.1%), and 50–65 years (23.1%). Of these, 61.1% identified as women, 38.3% as men, and 0.6% as non-binary. The vast majority of participants held at least a bachelor’s degree (80.6%) and demonstrated intermediate or higher proficiency in the English language (84.7%). Regarding their willingness to participate in a future corporate wellness intervention, 74.3% expressed a positive interest. Among them, 61.5% were motivated by goals of self-improvement or stress management. Additionally, the most preferred format for the intervention was a hybrid approach, combining online and onsite sessions (36%) (Figure 1, Figure 2 and Figure 3). These demographic findings indicate a well-educated workforce that values self-improvement and stress management. The preference for a hybrid intervention underscores the importance of flexible wellness solutions to accommodate diverse schedules and work environments.

### 3.2. Correlations and Structural Equation Model Analysis

As shown in Table 1, a Pearson correlation analysis revealed significant positive relationships among the variables of mindfulness (MAAS15), resilience (CDRISC10), and work ability (WAI). Mindfulness demonstrated statistically significant correlations with resilience (r = 0.389, *p* < 0.01) and work ability (r = 0.371, *p* < 0.01), indicating that individuals with higher mindfulness tend to exhibit greater resilience and better work ability. Resilience, in turn, showed a significant positive correlation with work ability (r = 0.408, *p* < 0.01), underscoring its critical role in enhancing workplace outcomes. Adherence to the Mediterranean lifestyle (MEDLIFE) was found to have lower but significant positive correlations with resilience (r = 0.170, *p* < 0.01) and work ability (r = 0.095, *p* < 0.05), suggesting that even moderate adherence to this lifestyle can contribute to these attributes. However, the relationship between the Mediterranean lifestyle and mindfulness was non-significant (r = 0.076, *p* > 0.05).

**Figure 4:**
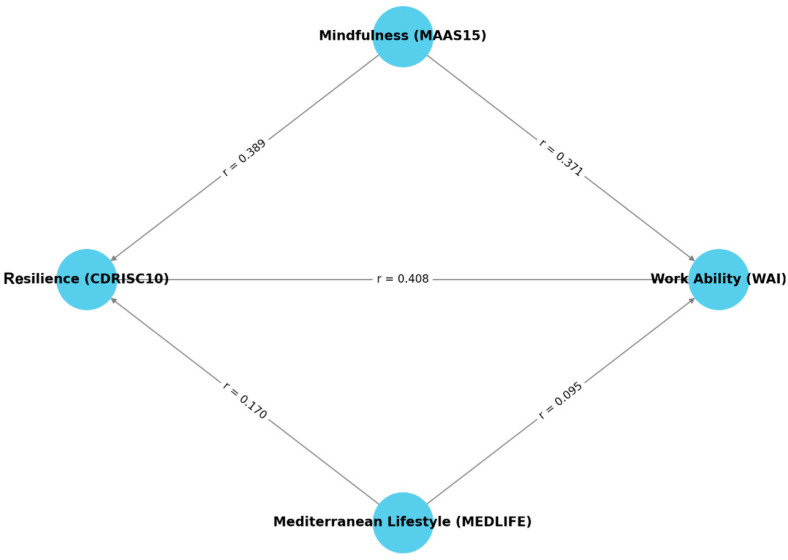
SEM Diagram presenting the relationships between Mindfulness, Resilience, Work Ability, and Mediterranean Lifestyle.

The Structural Equation Model (SEM) analysis further confirmed these relationships, highlighting the significant pathways among the variables. As shown in Figure 4, mindfulness directly influenced both resilience and work ability, with path coefficients of 0.389 (*p* < 0.01) and 0.371 (*p* < 0.01), respectively. Resilience also significantly contributed to work ability, with a path coefficient of 0.408 (*p* < 0.01), reinforcing its role as a key mediator in workplace outcomes. Adherence to the Mediterranean lifestyle demonstrated a positive but weaker influence on resilience (path coefficient = 0.170, *p* < 0.01) and work ability (path coefficient = 0.095, *p* < 0.05). However, no significant direct relationship was identified between mindfulness and the Mediterranean lifestyle (r = 0.076, *p* > 0.05).

These findings underscore the synergistic interaction among mindfulness, resilience, and adherence to the Mediterranean lifestyle in fostering work ability. While mindfulness and resilience exhibit particularly strong connections to work outcomes, the Mediterranean lifestyle provides an additional, albeit less impactful, contribution. From a practical perspective, these results suggest that corporate wellness programs focusing on enhancing mindfulness and resilience could yield substantial improvements in employee work ability. While adherence to the Mediterranean lifestyle plays a supportive role, it may be less immediately impactful for workplace interventions. Unexpectedly, the Mediterranean lifestyle did not show a significant correlation with mindfulness. This finding may indicate that lifestyle adherence alone does not necessarily enhance the cognitive or emotional traits associated with mindfulness.

The strongest correlation was between resilience and work ability (path coefficient = 0.408, *p* < 0.01), surpassing the direct effects of mindfulness and the Mediterranean lifestyle on work ability. Notably, mindfulness and the Mediterranean lifestyle exhibited stronger correlations with resilience than with work ability, suggesting that their impact on work outcomes may primarily operate through the enhancement of psychological resilience and, by extension, mental health. Further investigation could explore whether specific aspects of the Mediterranean lifestyle, such as social engagement or stress management practices, interact differently with mindfulness.

### 3.3. Mindfulness and Psychological Resilience

**Figure 5:**
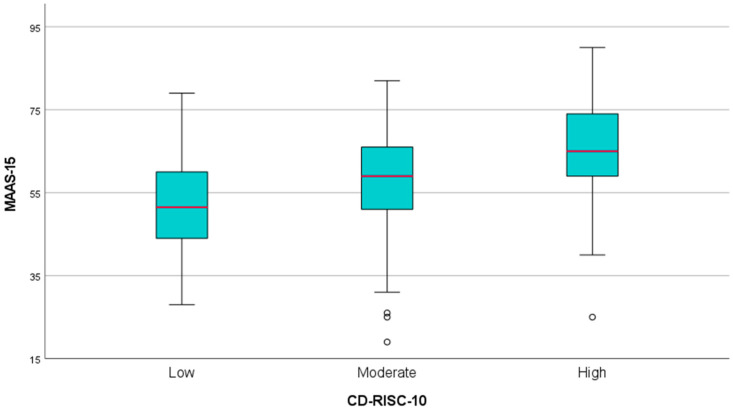
Boxplot Analysis: Mindfulness Scores Across Different Levels of Resilience.

A boxplot analysis was conducted to evaluate the relationship between resilience, as measured by the CD-RISC-10, and mindfulness, as assessed by the MAAS-15 scale (Figure 5). Participants were categorized into low, moderate, and high resilience groups based on their CD-RISC-10 scores. The results indicate a clear positive trend between resilience and mindfulness. Participants in the low resilience group had lower median MAAS-15 scores compared to those in the moderate and high resilience groups. Specifically, the interquartile range (IQR) for mindfulness scores was narrowest for the low resilience group, suggesting less variability in mindfulness among participants with low resilience. The moderate resilience group demonstrated higher median mindfulness scores and a slightly wider IQR, indicating a broader range of mindfulness levels in this group. The high resilience group exhibited the highest median mindfulness scores, with an even greater spread of values compared to the other groups. Outliers were observed in both the moderate and high resilience groups, but these did not substantially affect the overall trend. These results were further verified by a one-way ANOVA analysis, which confirmed significant differences in mindfulness scores across the three resilience groups (*p* < 0.01). This finding strengthens the evidence that higher levels of resilience are associated with higher levels of mindfulness, highlighting a potential predictive relationship between these variables.

### 3.4. Mindfulness and Job Satisfaction

**Figure 6:**
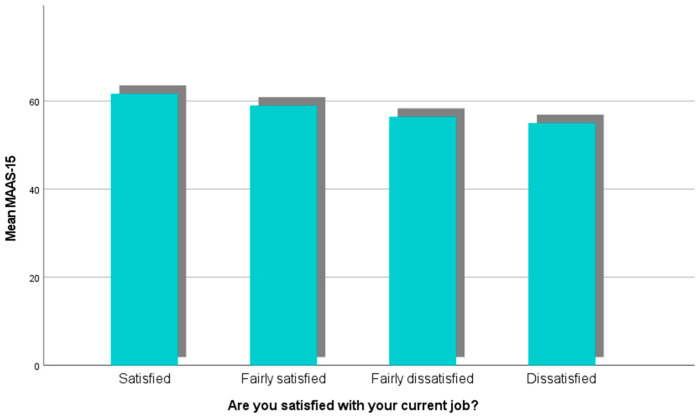
Means of Mindfulness across various levels of Job Satisfaction.

A one-way ANOVA was conducted to examine the relationship between mindfulness, as measured by the MAAS-15 scale, and job satisfaction, categorized into four groups: satisfied, fairly satisfied, fairly dissatisfied, and dissatisfied (Figure 6). The results showed a statistically significant difference in MAAS-15 scores across the four job satisfaction groups (F(3481) = 5.389, *p* = 0.001). This indicates that levels of mindfulness differ meaningfully depending on the degree of job satisfaction. Post hoc effect size analyses revealed that mindfulness explains 3.3% of the variance in job satisfaction (η^2^ = 0.033, 95% CI [0.006, 0.065]), which represents a small-to-moderate effect size. The epsilon-squared and omega-squared values (ε^2^ = 0.026, ω^2^ = 0.026) further confirmed the robustness of this finding, indicating that mindfulness accounts for approximately 2.6% of the variance in job satisfaction when adjusted for bias. These results suggest that higher levels of mindfulness, as reflected in the MAAS-15 scores, may be associated with higher levels of job satisfaction, emphasizing the potential role of mindfulness in fostering workplace well-being.

### 3.5. Mindfulness and Work Ability

**Figure 7:**
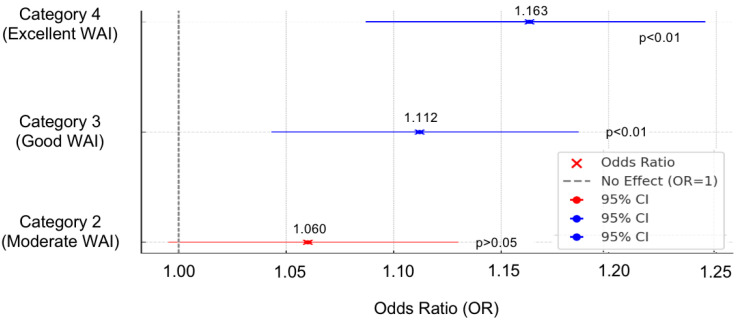
Odds Ratios of Mindfulness Across Various Work Ability Index Levels Compared to Poor Work Ability.

A multinomial logistic regression was conducted to examine the predictive role of mindfulness, measured by the MAAS-15 scale, in workplace well-being across different levels of the Work Ability Index, with category 1 being poor WAI, 2 moderate WAI, 3 good WAI, and 4 excellent WAI (Figure 7). The reference category for the analysis was poor working ability (category 1). Mindfulness was found to be a significant predictor of good and excellent working ability. For good working ability (category 3), mindfulness showed a significant positive association. The odds ratio was 1.112, with a 95 percent confidence interval of 1.043 to 1.186, and the *p*-value was 0.001. This indicates that for each one-unit increase in the MAAS15 score, the odds of having good working ability compared to poor working ability increased by approximately 11.2 percent. For excellent working ability (category 4), mindfulness demonstrated an even stronger predictive association. The odds ratio was 1.163, with a 95 percent confidence interval ranging from 1.087 to 1.245, and the *p*-value was less than 0.001. This suggests that a one-unit increase in the MAAS15 score was associated with a 16.3 percent increase in the odds of having excellent working ability compared to poor working ability. The significant intercept for excellent working ability (category 4) further highlights distinct differences in this group, reinforcing the predictive role of mindfulness in achieving higher levels of workplace well-being and productivity.

### 3.6. Mediterranean Lifestyle and Psychological Resilience

**Figure 8:**
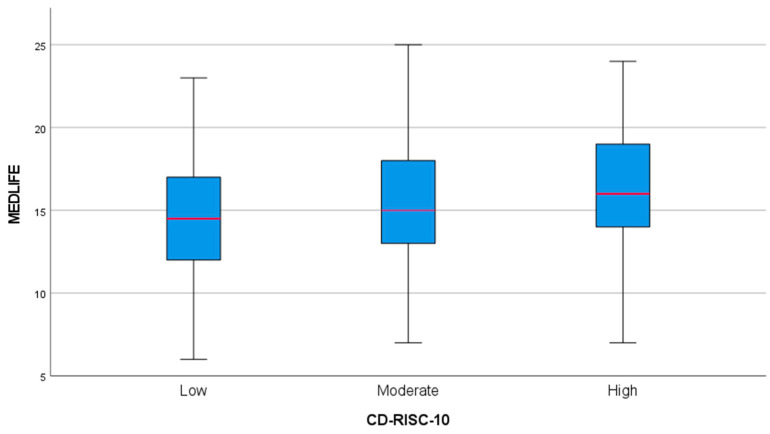
Boxplot analysis: Mediterranean Lifestyle across different levels of Resilience.

Psychological resilience, measured by the CD-RISC-10, showed a strong positive association with adherence to the Mediterranean lifestyle, as assessed by the MEDLIFE scale. A boxplot analysis revealed that participants with higher resilience had significantly greater adherence (Figure 8). The low resilience group displayed the lowest median MEDLIFE scores and minimal variability, as reflected by the narrowest interquartile range (IQR). The moderate resilience group exhibited higher median scores and a broader IQR, while the high resilience group demonstrated the highest median scores and the greatest variability. These findings were further validated by a one-way ANOVA, which confirmed significant differences in MEDLIFE scores across resilience levels (*p* < 0.01), emphasizing the robust relationship between resilience and the Mediterranean lifestyle.

### 3.7. Mediterranean Lifestyle and Work Ability

**Figure 9:**
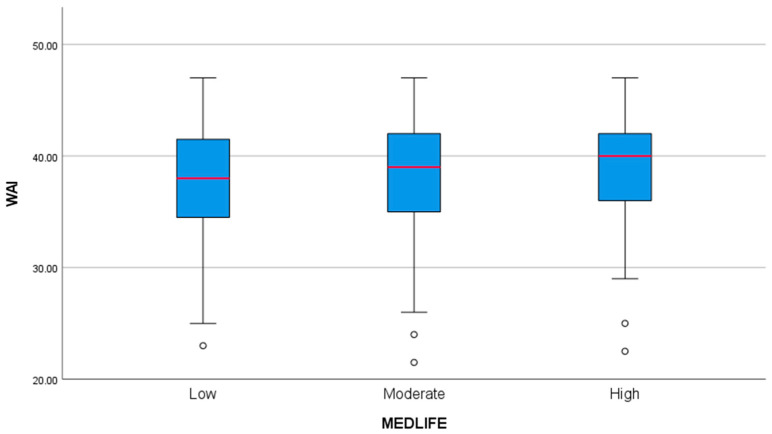
Boxplot analysis: Work Ability Index in various levels of Mediterranean Lifestyle.

A boxplot analysis was conducted to visualize the relationship between MEDLIFE adherence categories and the Work Ability Index (Figure 9). The analysis revealed a trend, with participants who demonstrated higher adherence to the Mediterranean lifestyle consistently reporting higher WAI scores. In order to explore further this trend, an ANOVA analysis was conducted to examine the relationship between adherence to the Mediterranean lifestyle (low, moderate, high) and the Work Ability Index. While the overall ANOVA result approached significance (F = 2.618, *p* = 0.074), small effect sizes, such as eta-squared (η^2^ = 0.011), indicated that 1.1% of the variance in work ability could be attributed to differences in Mediterranean lifestyle adherence. Post hoc LSD tests revealed that participants with high adherence (category 3) had significantly higher WAI scores compared to those with low adherence (category 1), with a mean difference of 1.398 (*p* = 0.024, 95% CI: 0.183 to 2.613). These findings suggest that higher adherence to the Mediterranean lifestyle may positively influence work ability, though the overall relationship is modest and warrants further investigation.

### 3.8. Mediterranean Lifestyle and Body Mass Index

An ANOVA analysis was conducted to examine the relationship between adherence to the Mediterranean lifestyle and Body Mass Index (BMI) categories: underweight, normal weight, overweight, and obesity. The results revealed a highly significant difference in BMI scores across Mediterranean lifestyle adherence groups (F = 364.764, *p* < 0.001). Effect size estimates indicated a strong association between the Mediterranean lifestyle and BMI, with Eta-squared = 0.695 (95% CI: 0.652–0.727) and similar values for Epsilon-squared and Omega-squared. Multinomial logistic regression analysis further explored the link between adherence to the Mediterranean lifestyle and Body Mass Index (BMI), using obesity as the reference category. The findings revealed that higher MEDLIFE scores were significantly associated with being in the normal weight category compared to obesity (B = 0.082, *p* = 0.020; Exp(B) = 1.086, 95% CI: 1.013 to 1.164), indicating that each one-point increase in MEDLIFE raised the odds of being in the normal weight category by approximately 8.6%. While there was a positive trend for the overweight category, it did not reach significance, and no significant association was observed for the underweight category. These results suggest that the Mediterranean lifestyle may play a key role in promoting healthier weight.

### 3.9. Work Ability and Other Wellness-Related Parameters

**Figure 10:**
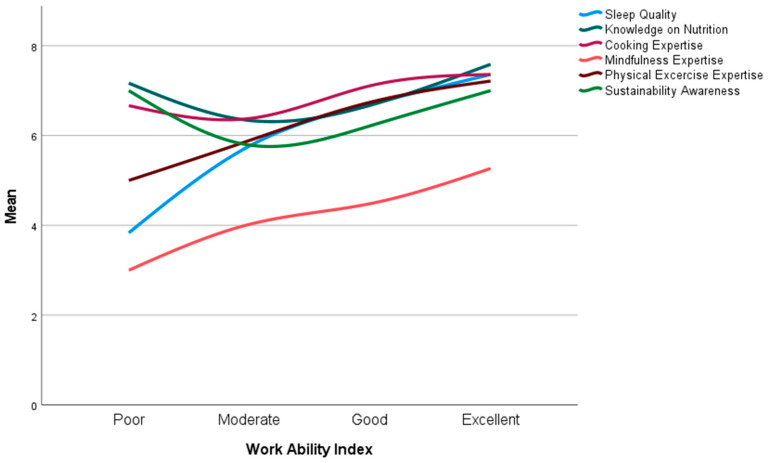
Means of wellness-related parameters in various Work Ability Index levels.

An ANOVA analysis examined the relationship between work ability and various wellness-related parameters (Figure 10). Significant differences were found in work ability across participants’ self-reported sleep quality (F = 12.506, *p* < 0.001), knowledge of human nutrition (F = 6.999, *p* < 0.001), expertise in meal preparation (F = 3.386, *p* = 0.018), mindfulness practices (F = 4.232, *p* = 0.006), physical exercise knowledge and experience (F = 7.959, *p* < 0.001), and awareness of the environmental impact of food production (F = 4.239, *p* = 0.006). Notably, the results for expertise in meal preparation, human nutrition, and sustainability followed a U-shaped distribution, indicating that both lower and higher levels of knowledge were associated with higher work ability, while moderate levels showed a weaker association. In contrast, expertise in mindfulness, physical exercise, and sleep quality exhibited a linear trend, with higher levels consistently correlating with greater work ability. These findings highlight the complex and varying influence of wellness factors on work ability.

### 3.10. Corporate Wellness and Work Sector

**Figure 11:**
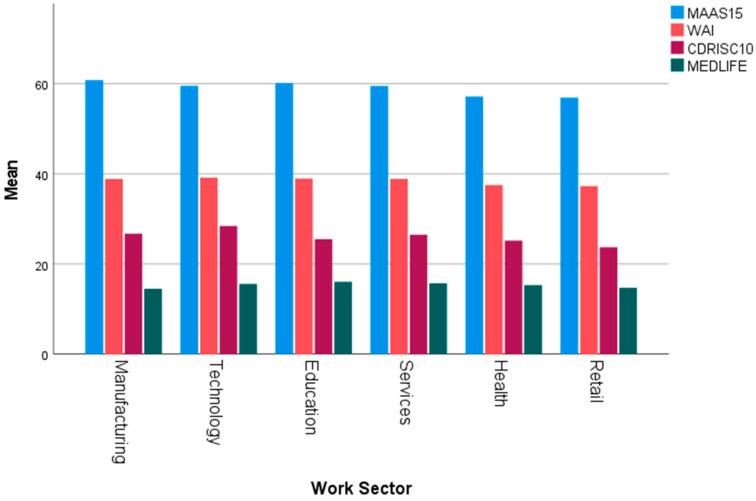
Means of Mindfulness, Work Ability, Resilience, and Mediterranean Lifestyle, across various work sectors.

A one-way ANOVA revealed a statistically significant difference in resilience levels (CD-RISC-10) across work sectors (*p* < 0.05), with employees in the health and retail sectors demonstrating the lowest mean resilience scores compared to other sectors. Similarly, LSD post hoc analysis identified significantly lower Work Ability Index (WAI) scores among employees in the health and retail sectors, reflecting diminished work ability in these groups. Furthermore, the LSD analysis revealed significantly lower mindfulness levels among health and retail employees compared to their counterparts in other sectors. In contrast, resilience, work ability, and mindfulness scores were relatively higher in sectors such as technology, education, and manufacturing. These results highlight sector-specific disparities in workplace wellness parameters, indicating the need for targeted strategies to address these gaps (Figure 11).

## 4. Discussion

This study explored the feasibility and impact of integrating mindfulness and adherence to the Mediterranean lifestyle as components of a corporate wellness framework, examining their associations with resilience, work ability, and job satisfaction. The findings underscore the potential of these factors to enhance workplace well-being, while also highlighting demographic and sector-specific nuances that merit further consideration.

The demographic analysis revealed a diverse workforce sample spanning multiple age groups, educational backgrounds, and levels of professional experience. The strong representation of participants with higher educational attainment (80.6% holding at least a bachelor’s degree) reflects a workforce likely to be receptive to wellness initiatives involving mindfulness and lifestyle modifications. Additionally, the significant interest expressed by participants in engaging with future corporate wellness programs (74.3%), particularly through hybrid formats combining online and onsite sessions, demonstrates both the feasibility and the demand for accessible and flexible intervention models. This willingness is particularly encouraging for organizations seeking to implement scalable wellness programs, as hybrid formats may offer practical solutions for engaging large and geographically dispersed employee populations.

The significant correlations between mindfulness, resilience, and work ability reaffirm their interdependence as critical components of workplace wellness. Higher mindfulness levels were strongly associated with greater resilience and improved work ability, indicating that mindfulness may serve as a foundational element for promoting both mental and physical occupational well-being. These findings are consistent with existing research demonstrating the role of mindfulness in reducing stress, enhancing emotional regulation, and improving cognitive functioning in professional settings [38,39]. The ability of mindfulness to predict superior job satisfaction and higher work ability scores further highlights its value as a key target for corporate wellness interventions.

Adherence to the Mediterranean lifestyle also emerged as an important contributor to workplace outcomes. Participants with higher MEDLIFE scores demonstrated better resilience, healthier BMI, and, to a lesser extent, improved work ability. These findings align with prior studies linking adherence to the Mediterranean diet and lifestyle with reduced cardiovascular risk, improved metabolic health, and better cognitive functioning [40,41]. Although the relationship between MEDLIFE and mindfulness was not significant, the positive association with resilience suggests that dietary and lifestyle practices could complement mindfulness in promoting overall well-being. The observed differences in BMI across adherence levels highlight the potential of the Mediterranean lifestyle to mitigate health risks associated with obesity, which is often linked to reduced productivity and higher absenteeism rates in the workplace [42].

The study also explored broader health-related factors influencing work ability and productivity. Sleep quality, expertise in mindfulness and physical exercise, meal preparation, human nutrition, and awareness of food sustainability were all significantly associated with work ability. Notably, a linear trend emerged for mindfulness, sleep quality, and physical exercise, with higher levels of these factors consistently linked to greater work ability. These results corroborate evidence suggesting that mental strength, physical fitness, and adequate sleep are essential for sustaining high levels of productivity and reducing workplace fatigue [43,44]. In contrast, the U-shaped distribution observed for nutrition and sustainability knowledge highlights the complexity of these relationships, suggesting that overly simplistic or excessively detailed knowledge in these areas may not always translate to optimal workplace outcomes.

Sector-specific disparities in wellness parameters further emphasize the need for targeted interventions. Employees in health and retail sectors reported significantly lower resilience, mindfulness, and work ability compared to those in technology, education, and manufacturing. These findings align with previous literature highlighting the disproportionate stress and burnout risks faced by frontline and service-oriented workers [45,46]. Tailored strategies addressing the unique demands of these sectors, such as mindfulness-based stress reduction programs or resilience training, could help mitigate these disparities and promote equitable outcomes across industries.

Despite the promising findings, this study is subject to several limitations. First, the cross-sectional design inherently restricts our ability to draw causal conclusions between mindfulness, the Mediterranean lifestyle, and workplace outcomes. Without a temporal framework, we cannot determine the directionality or causality of these relationships, making it impossible to ascertain whether these factors lead to improved workplace outcomes or are instead influenced by them. Longitudinal or experimental studies are essential to address this gap and establish the causal dynamics at play. Second, the sample was predominantly drawn from Greece and Cyprus, which raises concerns about the generalizability of the findings to different cultural contexts. The unique cultural and socio-economic characteristics of these regions may limit the applicability of the results to other populations. Future research should strive to include more diverse and representative samples to validate these findings in a broader array of organizational and cultural settings. Lastly, the reliance on self-reported data introduces significant potential for bias. Participants’ responses are prone to social desirability effects, where they may report behaviors or attitudes they believe are socially acceptable or expected, as well as recall biases, where they may inaccurately remember or misreport their experiences. These biases undermine the validity of the data and may have influenced the observed outcomes. Therefore, future studies should consider alternative data collection methods, such as objective measurements or third-party assessments, to mitigate these concerns [47].

Overall, the findings suggest that workplace interventions aiming to enhance well-being and productivity should adopt a holistic approach, addressing not only mindfulness and resilience but also broader health-related factors such as diet, physical activity, and sleep quality. Notably, the analysis highlights that the mechanism linking mindfulness and adherence to the Mediterranean lifestyle with work ability may operate primarily through the enhancement of psychological resilience, underscoring its pivotal role in fostering workplace outcomes. This multifaceted strategy would ensure a comprehensive enhancement of employee wellness, ultimately contributing to better organizational outcomes.

## 5. Conclusions

This study demonstrates the significant potential of mindfulness and adherence to the Mediterranean lifestyle (MEDLIFE) in enhancing workplace well-being, with positive correlations observed between mindfulness, resilience, and work ability. Participants with higher mindfulness exhibited greater resilience and better work ability, suggesting mindfulness’s crucial role in occupational outcomes. While the Mediterranean lifestyle showed a weaker association, it was linked to improved resilience and healthier BMI, underscoring the value of diet and lifestyle in supporting employee health and productivity. Factors such as sleep quality, physical exercise, and nutritional awareness were strongly associated with work ability, highlighting the importance of comprehensive wellness programs addressing both mental and physical health. Notably, the mechanism linking mindfulness and the Mediterranean lifestyle to work ability appears to operate primarily through their positive impact on psychological resilience, highlighting resilience as a pivotal intermediary in achieving better workplace outcomes.

To implement these findings, companies should develop hybrid wellness programs integrating mindfulness training, Mediterranean lifestyle education, and sector-specific resilience initiatives. Tailored programs and incentives can enhance engagement, particularly in industries like healthcare and retail, where wellness scores were lower. Longitudinal studies and randomized controlled trials are needed to validate these results and assess the long-term impact of these interventions. Future research should also explore how these approaches can be adapted for diverse industries and demographics, ensuring cultural and organizational alignment.

These findings underscore the feasibility and effectiveness of integrating mindfulness and Mediterranean lifestyle practices into corporate wellness strategies, promoting employee well-being, productivity, and job satisfaction.

## Figures and Tables

**Table 1:** Correlations among study variables.

	MAAS-15	MEDLIFE	CD-RISC-10	WAI
MAAS-15	1	0.076	0.389 **	0.371 **
MEDLIFE	0.076	1	0.170 **	0.095 *
CD-RISC-10	0.389 **	0.170 **	1	0.408 **
WAI	0.371 **	0.095 *	0.408 **	1

* Correlation is significant at the *p* < 0.05 level (2-tailed), ** Correlation is significant at the *p* < 0.01 level (2-tailed).

## Data Availability

The data presented in this study are available upon request from the corresponding author.
